# GFP-Fragment Reassembly Screens for the Functional Characterization of Variants of Uncertain Significance in Protein Interaction Domains of the *BRCA1* and *BRCA2* Genes

**DOI:** 10.3390/cancers11020151

**Published:** 2019-01-28

**Authors:** Laura Caleca, Mara Colombo, Thomas van Overeem Hansen, Conxi Lázaro, Siranoush Manoukian, Michael T. Parsons, Amanda B. Spurdle, Paolo Radice

**Affiliations:** 1 Unit of Molecular Bases of Genetic Risk and Genetic Testing, Department of Research, Fondazione IRCCS Istituto Nazionale dei Tumori, 20133 Milan, Italy; mara.colombo@istitutotumori.mi.it (M.C.); paolo.radice@istitutotumori.mi.it (P.R.); 2 Center for Genomic Medicine, Copenhagen University Hospital, Rigshospitalet, 2100 Copenhagen, Denmark; Thomas.Van.Overeem.Hansen@regionh.dk; 3 Department of Clinical Genetics, Copenhagen University Hospital, Rigshospitalet, 2100 Copenhagen, Denmark; 4 Hereditary Cancer Program, Catalan Institute of Oncology. Program in Molecular Mechanisms and Experimental Therapy in Oncology (Oncobell), IDIBELL, Hospitalet de Llobregat, 08900 Barcelona, Spain; conxi.lazaro@gmail.com; 5 Centro de Investigación Biomédica en Red de Cáncer (CIBERONC), 28029 Madrid, Spain; 6 Unit of Medical Genetics, Department of Medical Oncology and Hematology, Fondazione IRCCS Istituto Nazionale dei Tumori, 20133 Milan, Italy; Siranoush.Manoukian@istitutotumori.mi.it; 7 Department of Genetics and Computational Biology, QIMR Berghofer Medical Research Institute, Brisbane 4029, Australia; Michael.Parsons@qimrberghofer.edu.au (M.T.P.); Amanda.Spurdle@qimrberghofer.edu.au (A.B.S.)

**Keywords:** hereditary breast/ovarian cancer, *BRCA1*, *BRCA2*, variant of uncertain significance, functional assays, protein-protein interaction

## Abstract

Genetic testing for *BRCA1* and *BRCA2* genes has led to the identification of many unique variants of uncertain significance (VUS). Multifactorial likelihood models that predict the odds ratio for VUS in favor or against cancer causality, have been developed, but their use is conditioned by the amount of necessary data, which are difficult to obtain if a variant is rare. As an alternative, variants mapping to the coding regions can be examined using in vitro functional assays. BRCA1 and BRCA2 proteins promote genome protection by interacting with different proteins. In this study, we assessed the functional effect of two sets of variants in *BRCA* genes by exploiting the green fluorescent protein (GFP)-reassembly in vitro assay, which was set-up to test the BRCA1/BARD1, BRCA1/UbcH5a, and BRCA2/DSS1 interactions. Based on the findings observed for the validation panels of previously classified variants, BRCA1/UbcH5a and BRCA2/DSS1 binding assays showed 100% sensitivity and specificity in identifying pathogenic and non-pathogenic variants. While the actual efficiency of these assays in assessing the clinical significance of BRCA VUS has to be verified using larger validation panels, our results suggest that the GFP-reassembly assay is a robust method to identify variants affecting normal protein functioning and contributes to the classification of VUS.

## 1. Introduction

Genetic testing for the breast cancer susceptibility genes, *BRCA1* (MIM# 113705) and *BRCA2* (MIM# 600185), has become part of routine clinical care for patients with a personal or family history indicative of Hereditary Breast/Ovarian Cancer Syndrome (HBOC). A recent prospective study estimates the average cumulative risks by age 70 years of *BRCA1* carriers to be 60% and 59% for breast and ovarian cancer, respectively. For *BRCA2* carriers, the corresponding values are 55% and 16.5%, respectively [1]. Most pathogenic variants identified in the *BRCA1* and *BRCA2* genes are nonsense or frameshift that, by introducing a premature termination codon, lead to non-functional proteins. Splice site variants with a similar effect on the protein have also been identified [2]. In addition to these clearly loss-of-function alterations, a growing number of variants of uncertain significance (VUS) are currently being identified. These include missense substitutions, small in-frame deletions or insertions, alterations of both exonic and intronic sites potentially affecting splicing, and variants in regulatory sequences. The inability to classify the VUS either as disease causing or as rare non-pathogenic (benign) variation represents a challenge for genetic counselling, risk evaluation, and adoption of preventive and risk-reduction measures.

A five-tier classification scheme has been developed for the assessment of the clinical relevance of VUS by the International Agency for Research on Cancer (IARC) Working Group [3]. According to this scheme, Class 1 (posterior probability of pathogenicity < 0.001) and Class 2 (0.001 < posterior probability < 0.049) are considered non-pathogenic and likely non-pathogenic variants, Class 3 (0.05 < posterior probability < 0.949) VUS remain unclassified because of a lack of sufficient information for classification, Class 4 (0.95 < posterior probability < 0.99) and Class 5 (posterior probability > 0.99) are likely pathogenic and pathogenic variants, respectively. Posterior probability may be estimated using a multi-factorial model. For missense variants, this model combines a prior probability of causality, based on the amino acid evolutionary conservation model (A-GVGD, URL: http://agvgd.iarc.fr/) [4], with a likelihood ratio for causality derived from genetic and family-based data and histopathological tumor features [5,6,7,8]. However, since most *BRCA1* and *BRCA2* VUS are detected in a single or a few families only, this model is not able to classify the majority of them. Therefore, functional assays have been developed to independently classify VUS based on their effect on established functions of the BRCA1 and BRCA2 proteins [9,10].

Mounting evidence implicates both BRCA1 and BRCA2 proteins in all phases of the cell cycle and in the regulation of its orderly events [11] through their association with different proteins [12,13]. As a consequence, a defective protein-protein interaction may lead to uncontrolled cell replication and genomic instability, which are both hallmarks of tumor formation and progression. Thus, biochemical in vitro assays analyzing the formation of protein complexes provide a context in which to assess the functional effect of the *BRCA1* [14,15] and *BRCA2* VUS [16,17,18].

One of the most important BRCA1-interacting proteins is BARD1 (MIM# 601593). Under physiological conditions, BRCA1 is prevalently found to be associated with BARD1. This interaction is mediated by the N-terminal RING-finger domains of both proteins [19], which suggests that the heterodimer is the physiological relevant form of BRCA1 and the two proteins require each other for most of their cellular functions. Several lines of evidence support this view: BRCA1-BARD1 binding appears to stabilize BRCA1 protein expression [20] and to increase its nuclear accumulation by masking the nuclear export sequence (NES) flanking the RING-finger domain [21]. The two proteins co-localize in nuclear dots during the S-phase of the cell cycle and in nuclear foci at sites of DNA damage [22,23,24,25]. Most importantly, the binding with the E2 ubiquitin-conjugating enzyme UbcH5a (MIM# 602961) enables the BRCA1-BARD1 complex to acquire an ubiquitin ligase activity, linked to BRCA1 tumor suppression function by regulating transcription and double stand break (DSB) repair [26,27,28]. This is consistent with the reported hypersensitivity to Ionizing Radiation (IR) and to agent crosslinking the DNA, such as Cisplatin or Mitomycin C (MMC), of cells carrying BRCA1 RING pathogenic variants [29,30,31].

Concerning BRCA2, the strong physical interaction with the small (70-residues) DSS1 peptide (MIM# 601285), through its highly conserved C-terminal DSS1/ssDNA Binding Domain (DBD), appears to be crucial for the maintenance of its stability and correct conformation [32,33], for the repair of DNA DSBs by homologous recombination (HR) and the formation of DNA damage induced RAD51 foci [34]. Recently, it was shown that the nuclear retention of BRCA2 is regulated by DSS1 interaction through the masking of a NES, such that variation impairing BRCA2-DSS1 binding mis-localizes BRCA2 to the cytoplasm [35].

In this study, we analyzed the effect of a group of VUS mapped to the *BRCA1* and *BRCA2* gene regions encoding the RING-finger domain of BRCA1 and the DBD of BRCA2 on the binding with the interacting proteins BARD1, UbcH5a, and DSS1, using the previously established GFP-fragment reassembly screening [15,36]. In this assay, the GFP is dissected into two fragments (NfrGFP and CfrGFP) that, when expressed together in *E. coli* bacterial cells, do not spontaneously reassemble into a fluorescence protein. However, if each of the two fragments of the GFP are individually fused to two interacting protein domains, this interaction can mediate the reassembly of the GFP in co-transformed bacteria with consequent cellular fluorescence. We evaluated the performance of these approaches in correctly discriminating between pathogenic and non-pathogenic analysis of a panel of variants classified according to the IARC 5-class model (Leiden Open Variation Database, URL: http://hci-exlovd.hci.utah.edu/variants.php). In addition, we verified the consistency of the VUS classification with the results of other published functional analyses investigating the same variants [14,30,37,38,39,40,41,42,43,44,45,46,47,48,49,50].

## 2. Results

### 2.1. Selection of the BRCA1 and BRCA2 Variants

**Figure 1:**
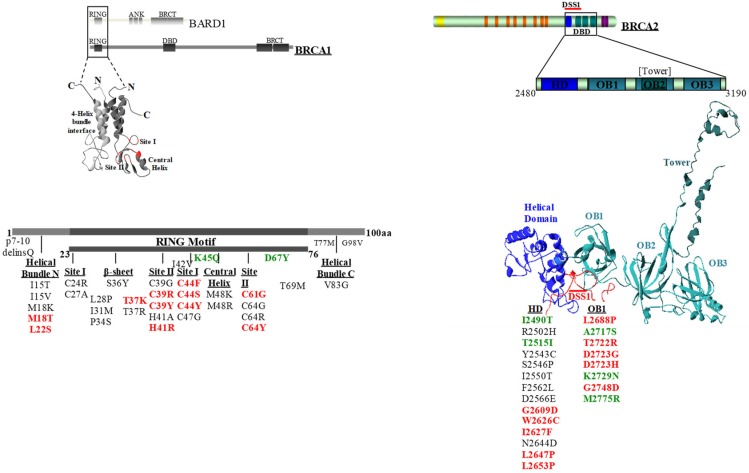
Diagram showing the location of specific motifs of the BRCA1 and BRCA2 proteins and the variants analyzed. Non-pathogenic variants (class 1 and class 2) are shown in green, pathogenic variants (class 4 and class 5) are shown in red, and VUS are shown in black. DBD, DNA binding domain. HD, helical domain. OB1, oligonucleotide/oligosaccharide binding fold 1. VUS, variants of uncertain significance.

We selected a total of 58 BRCA1 (*n* = 36) and BRCA2 (*n* = 22) variants mapping to the gene regions coding for the conserved motifs encompassing the RING-finger domain of BRCA1 and the DBD of BRCA2 [13] (Figure 1). Of these, 28 BRCA1 (*n* = 13) and BRCA2 (*n* = 15) variants, classified by the IARC-5 class system as pathogenic or non-pathogenic, were used for assay validation (Table 1). Thirty BRCA1 (*n* = 23) and BRCA2 (*n* = 7) variants were VUS (Table 2) of which 21 were identified among families referred for BRCA gene testing. Of the remaining nine variants, seven were extracted from the ClinVar database (https://www.ncbi.nlm.nih.gov/clinvar/), while two, p.Cys27Ala and p.His41Ala, were synthetic variants not yet observed in HBOC patients and were included so that at least one variant was examined for each of the critical cystein and histidine residues of the RING domain.

### 2.2. Evaluation of the Effect of BRCA1 Variants on the Interaction with the BARD1 and the UbcH5a Proteins by the GFP-Reassembly Assay

The N-terminal RING domain (aa 1–109) mediates the E3-ubiquitin ligase activity of BRCA1. It consists of a RING finger motif (aa 23–76) and two flanking α helices. It is characterized by a conserved pattern of seven cysteine and one histidine residues, which form two Zn^2+^-binding sites (termed Site I and Site II) stabilizing the RING structure. The RING finger motif assembles in a globular structure with three strand β-sheet and a central helix.

All 36 mutant constructs were co-transformed in bacterial cells with the pET11a-NfrGFP-BARD1 and pET11a-NfrGFP-UbcH5a constructs expressing the wild-type BARD1 (BD^N^) and UbcH5a (Ub^N^) proteins, which is in frame with the N-terminal fragment of the GFP protein, respectively. In addition, pET11a-NfrGFP-BARD1 and pET11a-NfrGFP-UbcH5a were co-transformed with pMRBAD-BRCA1-CfrGFP expressing wild-type BRCA1 (BR1^C^) and pMRBAD-Z-CfrGFP expressing the leucine zipper (Z) peptide (Z^C^), both in a frame with the C-terminal fragment of the GFP protein, as positive and non-cognate negative controls. The results are listed in Table 3.

**Figure 2:**
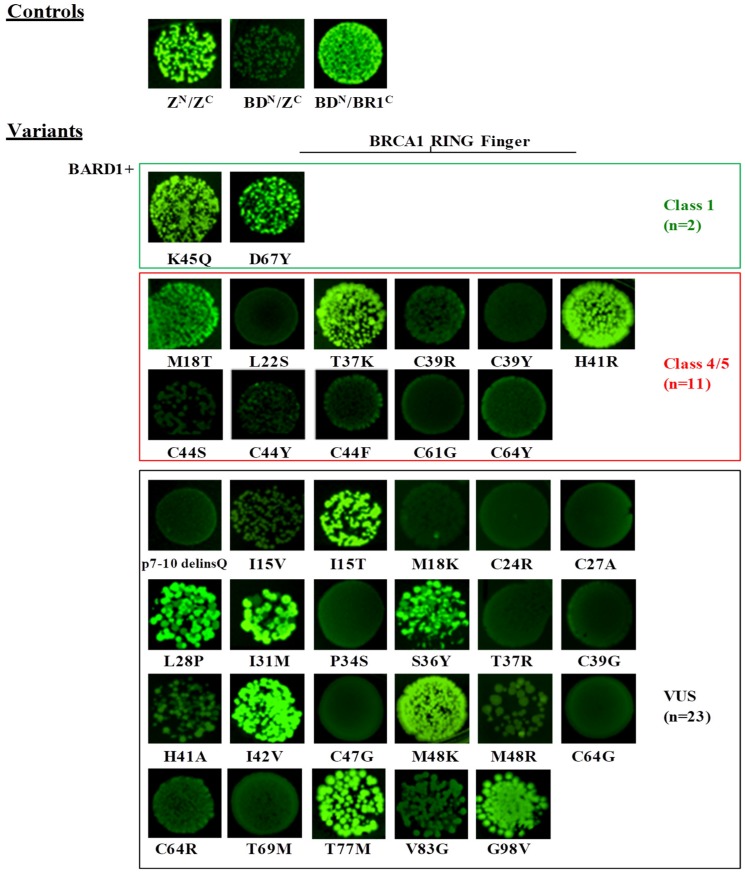
Detection of the BARD1/BRCA1 interaction by the GFP-fragment reassembly assay. Fluorescence was assessed after 24 h of growth at 30 °C followed by 2 days of incubation at RT. All pictures were taken with the same setting of digital camera (long-wave UV light, 365 nm). [Z^N^, H_6_-NfrGFPZ, Z^C^, ZCfrGFP, BD^N^, H_6_-NfrGFPBARD1, BR1^C^, BRCA1CfrGFP].

**Figure 3:**
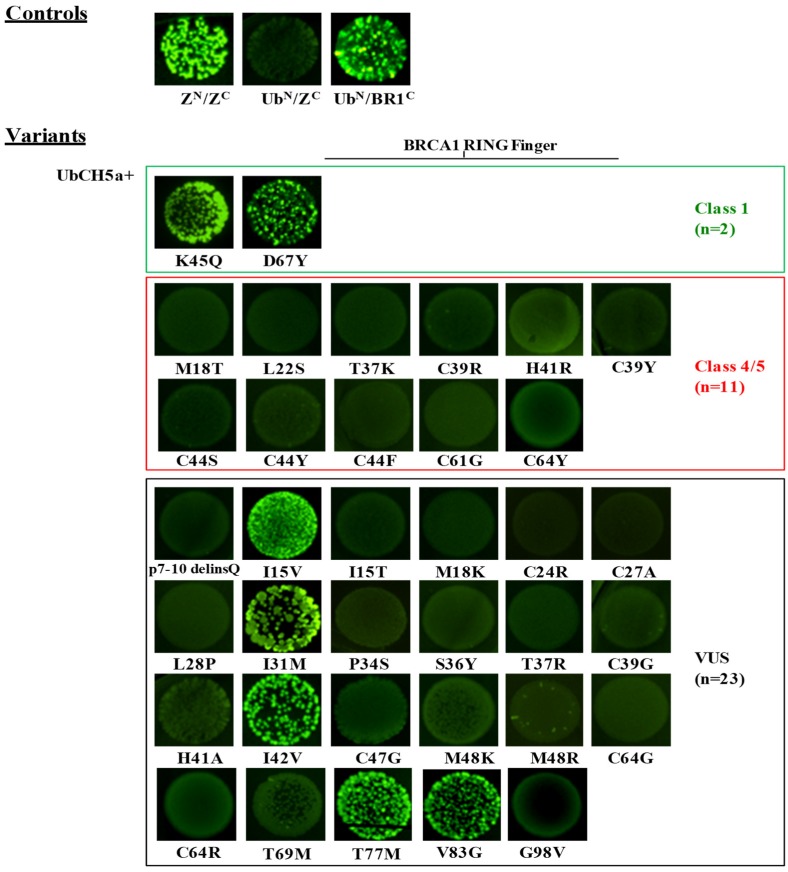
Detection of the UbcH5a/BRCA1 interaction by the GFP-fragment reassembly assay. Fluorescence was assessed after 24 h of growth at 30 °C followed by 2 days of incubation at RT. All pictures were taken with the same setting of digital camera (long-wave UV light, 365 nm). [Z^N^, H_6_-NfrGFPZ, Z^C^, ZCfrGFP, Ub^N^, H_6_-NfrGFPUbcH5a, BR1^C^, BRCA1CfrGFP].

Under inducing conditions, a bright fluorescence was observed in bacterial cells co-expressing the strong interacting anti-parallel leucine zipper NfrGFP-Z/Z-CfrGFP (Z^N^/Z^C^) fusion peptides and the positive controls (BD^N^/BR1^C^, Ub^N^/BR1^C^). No fluorescence was observed in bacterial cells co-expressing the non-cognate negative controls (BD^N^/Z^C^, Ub^N^/Z^C^) (Figure 2 and Figure 3). The following variants of the validation panel showed a fluorescence resembling that of the positive controls: p.Lys45Gln and p.Asp67Tyr (Class 1), p.Met18Thr (Class 4), p.Thr37Lys and p.His41Arg (Class 5), while no fluorescent signal was observed for the validation panel variants p.Leu22Ser, p.Cys39Arg, p.Cys39Tyr, p.Cys44Phe, p.Cys44Ser, p.Cys44Tyr, p.Cys61Gly and p.Cys64Tyr (all class 5). Among the analyzed VUS, p.Ile15Thr, p.Leu28Pro, p.Ile31Met, p.Ser36Tyr, p.Ile42Val, p.Met48Lys, p.Cys64Gly, p.Cys64Arg, p.Thr77Met, and p.Gly98Val showed bright fluorescence. Conversely, no fluorescence was observed in bacteria expressing the variants p.7-10delinsGln, p.Ile15Val, p.Met18Lys, p.Cys24Arg, p.Cys27Ala, p.Pro34Ser, p.Thr37Arg, p.Cys39Gly, p.His41Ala, p.Cys47Gly, p.Met48Arg, p.Cys64Tyr, p.Thr69Met., and p.Val83Gly (Figure 2).

The screening assessing the effect of BRCA1 variants on the binding to UbcH5a revealed bright fluorescence for bacterial cells co-expressing NfrGFP-UbcH5a together with BRCA1-CfrGFP bearing the class1 variants p.Lys45Gln and p.Asp67Tyr and the VUS p.Ile31Met, p.Ile42Val, p.Thr77Met, p.Ile15Val, and Val83Gly in the helical bundle interface (Figure 3). Conversely, all tested class 4 and class 5 variants were not able to bind to UbcH5a. These results indicate 100% sensitivity and specificity of this assay. All remaining tested VUS, including some of those that maintained the interaction with BARD1, namely p.Ile15Thr, p.Leu28Pro, p.Ser36Tyr, and p.Met48Lys, abrogated the binding with UbcH5a.

**Figure 4:**
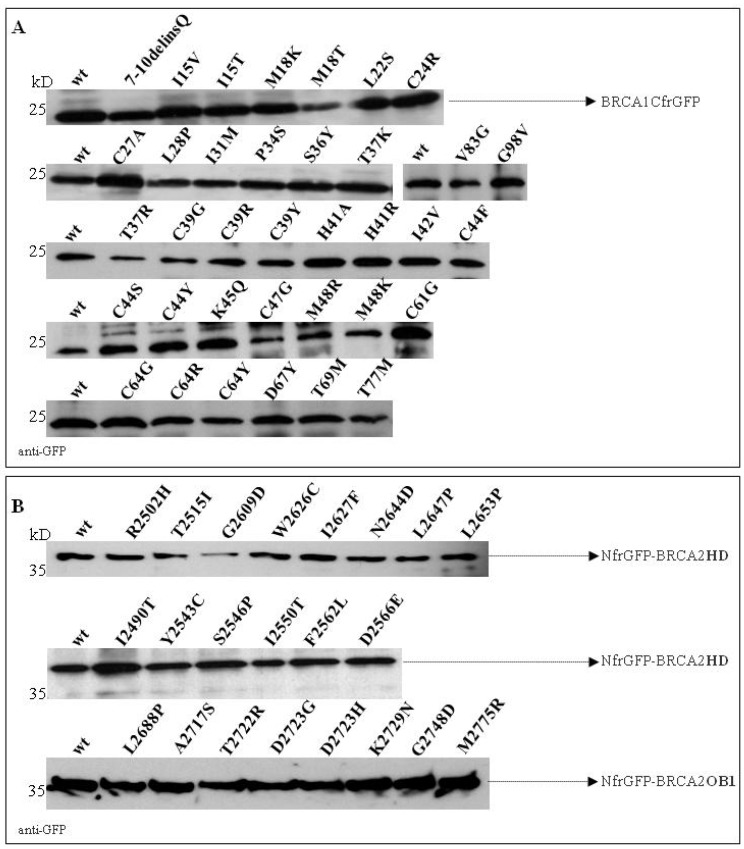
Expression of BRCA1-CfrGFP (**A**) and NfrGFP-BRCA2HD/OB1 (**B**) wild-type (wt) and mutant constructs. The molecular masses are indicated on the left.

To confirm that the loss of fluorescence was attributable to the lack of binding to BARD1 and/or UbcH5a and not to poor expression of the BRCA1 mutants, the cell lysates were visualized by Western blotting against the GFP tag. The results showed that all fusion peptides were expressed to a similar extent (Figure 4A).

### 2.3. Evaluation of the Effect of the BRCA2 Variants on the Interaction with the DSS1 Protein by the GFP-Reassembly Assay

The C-terminal region of BRCA2 contains a DSS1/ssDNA Binding Domain (DBD) consisting of five components. The first is described as the helical domain (HD) due to its 190 amino acids consisting mainly of α-helices. This is followed by three oligonucleotide/oligosaccharide binding folds (OB1, OB2 and OB3). In addition, within OB2, a 130 amino acid insertion exists, which was named the tower domain due to its tower-like structure protruding from the OB fold. The crystallographic study of BRCA2-DBD revealed a stoichiometric complex with DSS1, which is almost entirely mediated by highly conserved residues spanning the HD and OB1 motifs. Solely, one DSS1 residue interacts with the first portion of the OB2 motif [32].

**Figure 5:**
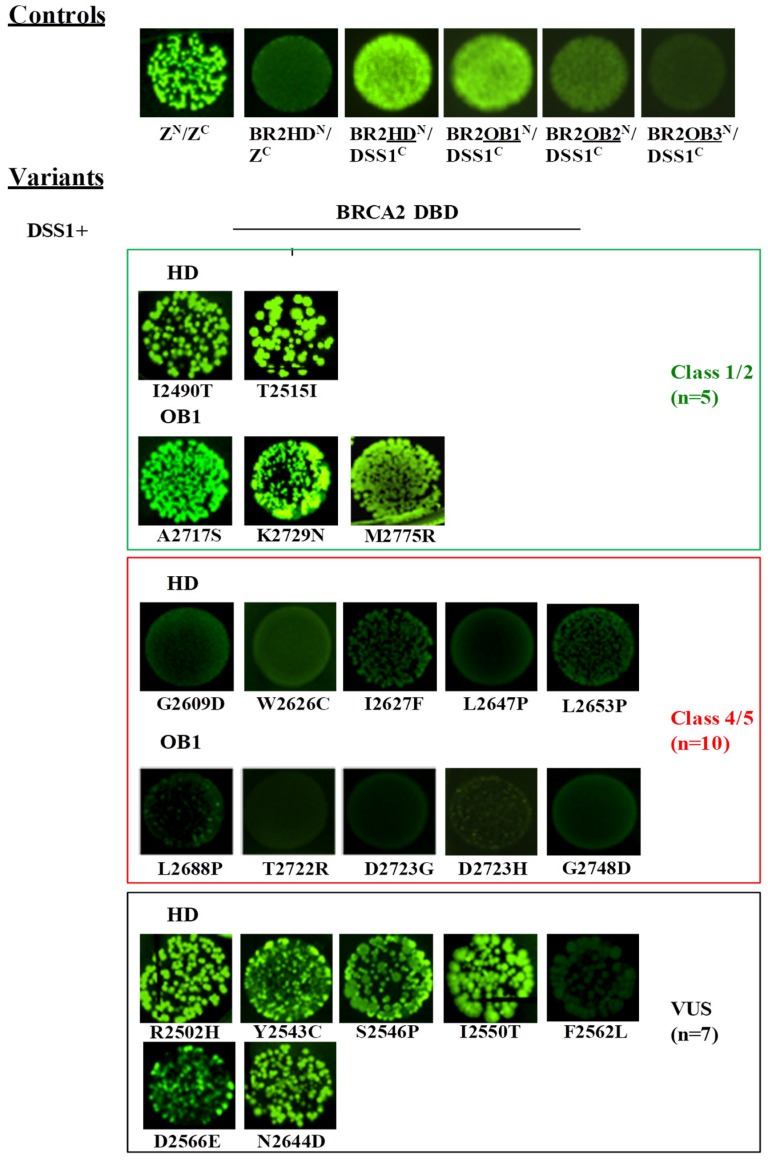
Detection of the DSS1/BRCA2 interaction by GFP-fragment reassembly assay. Fluorescence was assessed after 24 h of growth at 30 °C followed by 2 days of incubation at RT. All pictures were taken with the same setting of digital camera (long-wave UV light, 365 nm). [Z^N^, H_6_-NfrGFPZ, Z^C^, ZCfrGFP, BR2HD^N^, H_6_-NfrGFPBRCA2HD, BR2OB1^N^, H_6_-NfrGFPBRCA2OB1, BR2OB2^N^, H_6_-NfrGFPBRCA2OB2, BR2OB3^N^, H_6_-NfrGFPBRCA2OB3, DSS1^C^, DSS1CfrGFP].

We replaced the leucine zipper peptides in the original constructs (pET11a-NfrGFP-Z and pMRBAD-Z-CfrGFP) with HD, the three OB motifs, each of which is in a distinct construct and the protein DSS1 is in all the possible orientations (i.e., pET11a-NfrGFP-HD/OB1/OB2/OB3 pET11a-NfrGFP-DSS1, pMRBAD-HD/OB1/OB2/OB3-CfrGFP, and pMRBAD-DSS1-CfrGFP). Upon co-transformation in bacterial cells, bright fluorescence was observed only when the HD and OB1 were fused to the NfrGFP and DSS1 was fused to the CfrGFP while a less pronounced fluorescence was observed in bacterial cells co-expressing BRCA2-OB2 and DSS1 inserted in the same orientation (Figure 5). No fluorescence was detected when the above inserts were fused in the opposite orientation and co-transfected likely due to a conformational restraint of GFP-reassembly under these conditions. Consistently with the BRCA2 DBD-DSS1 crystal structure, no fluorescence was observed in bacteria co-expressing BRCA2-OB3 and DSS1 in either orientation (Figure 5 and Supplemental Figure S1).

Based on the above results, we decided to evaluate the effect of the *BRCA2* variants mapped to the gene regions encoding for the HD and OB1 motifs only. All 22 mutants and the corresponding wild-type forms were co-transformed with the pMRBAD-DSS1-CfrGFP expressing the wild-type DSS1. In addition, the pET11a-NfrGFP-HD was co-transformed with the pMRBAD-Z-CfrGFP expressing the leucine zipper peptide as a non-cognate negative control.

As reported in Figure 5, no fluorescence was observed in bacterial cells co-expressing non-cognate negative control NfrGFP-HD/Z-CfrGFP (HD^N^/Z^C^) while bright fluorescence was detected in bacteria co-expressing the positive controls Z^N^/Z^C^, NfrGFP-HD/DSS1-CfrGFP (HD^N^/DSS1^C^), and NfrGFP-OB1/DSS1-CfrGFP (OB1^N^/DSS1^C^). Comparable fluorescence was observed in bacterial cells co-expressing DSS1-CfrGFP with NfrGFP fused to the BRCA2 peptides bearing all the variants of Class 1/2 and the following VUS: p.Arg2502His, p.Tyr2543Cys, p.Ser2546Pro, p.Ile2550Thr, p.Asp2566Glu, and p.Asn2644Asp (HD). No fluorescence was observed in all the bacteria expressing BRCA2 peptides carrying the Class 4/5 mutations and the VUS p.Phe2562Leu (HD). The expression level of all the NfrGFP-HD/OB1 peptides was verified by Western blotting analysis using an anti-GFP antibody and no significant differences were observed (Figure 4B).

### 2.4. Validation of the GFP-Reassembly Screenings

The re-assembled complexes were purified by Immobilized Metal Affinity Chromatography (IMAC) from the soluble fraction of co-transformed bacterial cell lysates exploiting the hexahistidine tag (H_6_) fused to the N-terminus of the NGFP fragments [14,15]. Equal aliquots of the cleared lysates of co-transformed *E. coli* BL21 (DE3) cells were incubated with Ni-NTA agarose resin and the affinity-captured material was analyzed by Western blotting using a polyclonal anti-GFP antibody (Supplemental Figures S2–S4). The detection of two bands corresponding to the complex components confirmed interactions with the binding proteins for all BRCA1 and BRCA2 constructs that tested positive in the fluorescence reassembly assay. No bands, or bands of very low intensity, were observed in case BRCA1 and BRCA2 variants for whom no fluorescence was detected, which confirms the lack of interaction. Assay specificity was supported by the absence of signals detected in cell lysates co-expressing the NfrGFP-BARD1, NfrGFP-UbcH5a, or the NfrGFP-HD with the CfrGFP-Z peptide (non-cognate negative controls).

### 2.5. Evaluation of the Effect of VUS on mRNA Splicing

Exonic variants may affect protein functions not only by altering the amino acid sequence, but also abolishing naturally-occurring splice sites or introducing/activating new or cryptic splice sites. To account for this possibility, we performed an in silico analysis on all 30 VUS included in the study, from which only three were predicted to be spliceogenic by ≥three algorithms of the Alamut software. These included *BRCA1* c.110C>G and c.115T>G, predicted to introduce novel acceptor splice sites, and *BRCA1* c.190T>G, predicted to reinforce the strength of an alternatively used donor splice site in exon 5. Existing mRNA assay data reports no effect on mRNA splicing for *BRCA1* c.110C>G [57], while *BRCA1* c.190T>G has been shown to lead to increased levels of a non-productive naturally-occurring isoform (Δ5q) [58], which carries the out of frame deletion of the distal portion of exon 5 [59]. To the best of our knowledge, no experimental data has been reported as for the c.115T>G. No spliceogenic effect was predicted for all remaining VUS.

## 3. Discussion

The reporting of VUS can cause uncertainty and anxiety regarding cancer risk and clinical management. In addition, appropriate medical care may be delayed for individuals carrying VUS that are later determined to be pathogenic. In the near future, the relevance of these questions is likely to rise, as the relative frequency of VUS on the total number of variants identified in HBOC individuals is expected to increase dramatically with the development of next generation sequencing (NGS) analyses and the possibility to screen gene panels containing several verified or putative breast/ovarian cancer predisposition genes at minimal additional costs [60]. Furthermore, a study evaluating the coding regions and exon-intron boundaries of a 42-cancer gene sequencing panel (including *BRCA1* and *BRCA2*) identified one or more VUS in approximately 90% of the patients [61]. This underlines the need to develop accurate methods to discriminate VUS that are actually related to cancer risk (pathogenic) from those without clinical relevance (non-pathogenic or benign).

In this study, we evaluated the use of an in vitro protein-fragment complementation assay and the GFP-fragment reassembly screening for the functional characterization of *BRCA1* and *BRCA2* VUS. We had previously applied this method to functionally characterize an Italian founder variant (p.Cys64Arg) mapped to the RING-finger domain of BRCA1 [15]. This variant was shown to abolish the interaction of BRCA1 with the partner protein BARD1, in a manner similar to the known cancer predisposing variant p.Cys61Gly [62]. In this case, we applied the same approach to analyze the effect of 25 *BRCA1* VUS mapped to the RING-finger domain on the binding with BARD1 and UbcH5a proteins and seven *BRCA2* VUS mapped to the DBD domain on the binding with the DSS1 protein.

To evaluate the accuracy of the assays in discriminating pathogenic from non-pathogenic variants, we tested the effect of a panel of 13 *BRCA1* and 15 *BRCA2* variants (validation panels) previously classified according to the IARC 5-class system. The assays analyzing the BRCA1/UbcH5a and BRCA2/DSS1 interactions show 100% sensitivity (percentage of correctly classified pathogenic variants) and 100% specificity (percentage of correctly classified non-pathogenic variants). In fact, all constructs carrying the Class 1 and 2 variants included in the validation panels displayed complex formation with the selected binding partners, while all constructs carrying the Class 4 and 5 variants did not. Based on these findings, our investigation shows that 19 (18 *BRCA1* and 1 *BRCA2*) VUS behaved as pathogenic and 11 (5 *BRCA1* and 6 *BRCA2*) as neutral variants (Figure 3 and Figure 5, Table 3 and Table 4).

Two issues need to be considered when evaluating the performance of the GFP-fragment reassembly assay. First, since the assays described are based on an artificial experimental model, where functional domains of human proteins are expressed in prokaryotic cells, the behavior of mutant constructs that we observed might not reflect the properties of full-length BRCA proteins expressed in their ‘natural’ context. However, it must be considered that, although our observations might actually provide clues for the molecular mechanisms mediating the tumor suppressor activity of BRCA proteins (see below), the aim of this study was essentially to collect evidence for the clinical relevance of BRCA VUS by the use of functional assays that efficiently dichotomize variants into pathogenic or non-pathogenic. Second, some exonic non-synonymous changes can affect gene functionality by altering mRNA splicing. In a few cases, i.e., when the spliceogenic allele retains the ability to produce a full-length protein, this occurrence may co-exist with a pathogenic effect of the missense substitution such as in the case of the *BRCA2* c.8168A>G (p.Asp2723Gly) [47,56]. Previous data and in silico analyses revealed for two of the *BRCA1* VUS we tested, an actual (c.190T>G) or potential (c.115T>G) spliceogenic effect. Based on the outcomes of the BRCA/UbcH5a interaction assay, all the above variants were predicted to be pathogenic. However, no data are presently available to ascertain if full-length proteins carrying such variants are actually expressed, and, therefore, no data are present to hypothesize dual mRNA/protein defects.

The assay analyzing the BRCA1/BARD1 binding reveals high specificity (100%), but lower sensitivity (62.5%), compared to the assay exploiting BRCA1/UbcH5a interaction, which indicates that the analyses of different protein-protein interactions may have a different level of accuracy in predicting the pathogenicity of amino acid changes in binding domains. In fact, the class 4 p.Met18Thr and the class 5 p.Tyr37Lys and p.His41Arg variants preserved the interaction with BARD1 as the wild-type protein and the proteins carrying the Class1 variants p.Lys45Gln and p.Asp67Glu.

Similar results were observed using alternative approaches in independent studies (Table 3). Morris and co-workers reported a comprehensive analysis of missense mutations mapped to the RING-finger domain of BRCA1 and, using yeast 2-hybrid (Y2H) and in vitro ubiquitin ligase assays, they showed that, in the presence of the p.Met18Thr variant, BRCA1: (a) retains the interaction with BARD1, (b) is no longer able to bind UbcH5a, and (c) loses the E3 ubiquitin ligase activity [37]. Ransburgh and co-workers exploiting the co-immunoprecipitation approach showed that the variant p.Met18Thr only modestly affects the interaction between BRCA1 and BARD1 [38]. Moreover, a recent analysis measuring the effect of approximately 2000 *BRCA1* missense substitutions, both on BARD1 binding and E3 ubiquitin ligase activity, reported that the BRCA1 proteins carrying the variants p.Met18Thr, p.Thr37Lys, and p.His41Arg are able to interact with BARD1 and lose the E3 ligase activity [43]. All these observations, together with our results, suggest that, in the absence of the BRCA1-UbcH5a complex, the maintenance of the binding with BARD1 is dispensable for the BRCA1 tumor suppressor activity. Thus, we speculate that the reduced or abrogated E3 ubiquitin ligase activity, through loss of binding with UbcH5a, is correlated with disease susceptibility and the prediction of pathogenicity. The observations of Drost and co-workers support this hypothesis, who reported that mice carrying the pathogenic p.Cys61Gly variant, inactivating BRCA1 E3 ligase activity [37], are embryonically lethal because of a developmental delay and that, in addition, the latency and nature of mammary tumors with one *Brca1* allele carrying the p.Cys61Gly variant and one *Brca1* conditional null allele were similar to those carrying two Brca1 null alleles [63]. Consistently with these findings, two of the BRCA1 VUS that, in our analysis, did not inhibit the BARD1 interaction, but lose the UbcH5a, and for which data on enzymatic properties are available (p.Ile15Thr, p.Leu28Pro) and lack ubiquitin ligase activity [37] (Table 3). However, it has to be mentioned that, in another study, the missense substitution p.Ile26Ala, abrogating E2 binding and thus ubiquitin-ligase activity, was found to prevent tumor formation to the same degree as wild-type *Brca1* in three conditional genetic models. Furthermore, the DNA damage response remained intact with no changes in chromosome stability or sensitivity to genotoxic stress in mouse embryonic fibroblasts [64]. Given the discrepancy of the results from the two studies, the implication of BRCA1 E3 ligase activity in tumor suppression remains to be elucidated.

In the presence of p.Thr77Met, BRCA1 is able to bind both BARD1 and UbcH5a proteins. However, as reported by Morris and co-workers, the proteins lose the ubiquitin ligase activity [37]. A possible explanation is that a weak interaction between BRCA1 and UbcH5a is not sufficient to promote the ubiquitin ligase activity. Actually, the GFP-fragment reassembly approach can detect very weak and transient interactions and the fluorescence intensity observed does not necessarily correlate quantitatively to the interaction affinity of the proteins, which represents a limitation for this assay. Therefore, hypomorphic variants (i.e., variants weakening, but not completely disrupting, the interaction of BRCA1 and BRCA2 with partner binding proteins, which retains partial protein activity) are likely to produce a positive fluorescent signal and can be categorized as non-pathogenic with this approach. In effect, using the Y2H assay, Morris and co-workers showed a reduction in binding to the UbcH5a of BRCA1 carrying the p.Thr77Met variant. These observations suggest that the p.Thr77Met may be a hypomorphic variant associated with moderate-to-low risk of breast and ovarian cancers. Furthermore, this variant was observed in trans with a pathogenic variant in an individual without clinical signs of Fanconi anemia (FA) or FA-like disease, which is consistent with the suggestion that the p.Thr77Met is hypomorphic and is not associated with a high risk of cancer. Further analyses, using additional approaches such as the Y2H assay and the split biosensor assays measuring protein interaction kinetics (the tripartite split-GFP association assay [65]), are required to define the thresholds of binding activity that discriminate between pathogenic and neutral variants and assess potential intermediate effects.

A series of important considerations have to be applied to functional assays evaluating the *BRCA1* and *BRCA2* VUS [10,11]. In particular, a panel of different validated assays, representative of different protein functions, should be used, in order to avoid the misinterpretation of the clinical relevance of BRCA gene variants. We observed good correlation of results obtained from the GFP-reassembly screenings with those of other reported functional assays, including homology-directed repair (HDR), mouse embryo stem cells (mESCs) viability, and drug resistance assays (Table 3 and Table 4). In fact, *BRCA1* and *BRCA2* VUS that were proficient in these assays, retain binding activity with UbCH5a and DSS1, respectively. All variants that were not proficient in other reported assays showed a loss of the binding activities. These included the BRCA2 p.Phe2562Leu, which is the only VUS that, in our assay, abolishes the ability of BRCA2 DBD to bind DSS1, which is reported to be deficient in HDR activity [48].

To date, the most accurate medium throughput functional approaches in evaluating *BRCA1* and *BRCA2* VUS are the yeast-cell based colony size phenotype (SCP) assay [40,66] and the HDR assay in mammalian cells [47,48], respectively (Table 3 and Table 4). Compared to the GFP-reassembly screening, the SCP assay shows lower sensitivity (96%) and specificity (93%). Importantly, contrary to the SCP assay, the GFP-reassembly screening analyzing the effect of BRCA1 variants on UbcH5a binding correctly classifies the p.Cys61Gly variant as pathogenic (Table 3). While the GFP-reassembly assay and the HDR functional assay show the same level of accuracy, the latter is more technically and experimentally demanding and time consuming.

While this manuscript was in preparation, Findlay and co-workers published the results of a massive functional characterization of *BRCA1* single nucleotide variants, (SNVs) based on a highly sensitive and specific saturation genome editing (SGE) approach [44]. Findlay et al. measured the effect on the survival of the near-haploid HAP1 cell line of almost 4,000 SNVs corresponding to 96.5% of all possible base-pair changes in 13 exons that encode the RING and BRCT domains. The assay was based on the observation that the maintenance of *BRCA1*-mediated HDR is necessary for the viability of HAP1 cells. Notably, we observed a 100% correlation between the functional classification based on SGE scores and the outcomes of our GFP-reassembly assay evaluating the BRCA1/UbCH5a interaction (Table 3). This strict consistency suggests that BRCA1/UbCH5a interaction, which mediates BRCA1 ligase activity, is essential for proficient HDR. In addition, since HDR deficiency is targeted by drugs exploiting synthetic lethality, such as Poly (ADP-ribose) polymerase 1 (PARP1) inhibitors [67], our assay could be used to predict a response to therapeutic treatments.

SGE has demonstrated to be a viable and highly productive strategy for functionally classifying thousands of variants in clinically actionable genes such as *BRCA1*, including a large number of variants that, to date, have not been observed in humans. In fact, a major strength of this approach is represented by the possibility of examining variants located throughout the entire gene sequence, while, on the contrary, the GFP-reassembly assay may be applied only to gene regions coding for protein-interaction domains. However, it must be noted that the vast majority of pathogenic BRCA gene variants lie in these domains [6,51]. On the other hand, SGE analyses are limited by the extreme difficulty of generating all possible gene variants that may occur in patients. In particular, substitutions and insertions/deletions involve more than one nucleotide. For example, in our study, we could analyze three variants identified in families referred for BRCA gene testing, namely the *BRCA1* c.20_29del9 (p.7-10delinsGln) and c.206_207delCCinsTG (p.Thr69Met) in frame deletions, and the c.107C>A (p.Ser36Tyr) SNV, that were not evaluated in the high-throughput study of Findlay and co-workers.

This represents an important and attractive advantage of the split-GFP reassembly in vitro method, which, together with its easiness of use and robustness, makes it suitable for routine analyses of naturally occurring genetic variants. Moreover, this assay could be applied to large-scale studies that are required to achieve robust estimates of the accuracy of functional approaches for VUS classification and clinical applicability.

## 4. Materials and Methods

### 4.1. Variant Selection

All examined variants were observed in high risk HBOC families fulfilling the ascertainment criteria for BRCA1/2 testing in use at the collaborating institutions, including the Fondazione IRCCS Istituto Nazionale dei Tumori (Milan, Italy) [68], the Catalan Institute of Oncologia (Hospitalet de Llobregat, Barcelona, Spain) [69], and the Rigshospitalet (Copenhagen, Denmark) [70].

### 4.2. Plasmid Construction

The pET11a-NfrGFP-Z, pMRBAD-Z-CfrGFP, pET11a-NfrGFP-BARD1, and pMRBAD-BRCA1-CfrGFP expression vectors were obtained and generated as previously described [14]. The BRCA2 fragments encoding the Helical Domain (HD, amino acids 2480–2667), three oligonucleotide-binding folds (OB1, amino acids 2668–2807, OB2, amino acids 2808–3048, OB3, amino acids 3050–3185), the full-length coding sequences of the DSS1, and the UbcH5a genes were obtained by PCR amplification of cDNA prepared from 293T cells using a RevertAid H Minus First Strand cDNA Synthesis Kit (Thermo Scientific, Waltham, MA, USA), and cloned into pET11a-NfrGFP-Z between XhoI and BamHI restriction sites (HD, OB1, OB2, OB3, DSS1, and UbcH5a), and pMRBAD-Z-CfrGFP between NcoI and AatII restriction sites (HD, OB1, OB2, OB3, and DSS1) after removing the leucine zipper motifs (Z). All of the *BRCA1* and *BRCA2* variants were obtained by direct mutagenesis of pMRBAD-BRCA1-CfrGFP and of pET11a-NfrGFP-BRCA2HD/OB1 using the QuickChange XL Site-directed Mutagenesis Kit (Stratagene, La Jolla, CA, USA), according to the manufacturer’s instruction. DNA sequencing (Eurofins Genomics, Ebersberg, Germany) technology checked the recombinant clones.

### 4.3. Expression of Recombinant Peptides

*E. coli* BL21 (DE3) cells were transformed with pET11a-NfrGFP vector series or pMRBAD-CfrGFP vector series. Single colonies were picked and used to inoculate 20 mL of LB broth medium containing 100 µg/mL of ampicillin (pET11a-NfrGFP vector series, Addgene, Watertown, MA, USA) or 35 µg/mL of kanamycin (pMRBAD-CfrGFP vector series, Addgene, Watertown, MA, USA). The colonies were then incubated at 37 °C until the Optical Density measured at a wavelength of 600 nm (OD_600_) reached 0.6. Then, induction of recombinant peptides was carried out with the addition of IPTG to a final concentration of 20 µM (pET11a-NfrGFP vector series) and 0.2% of L-arabinose (pMRBAD-CfrGFP vector series). After growing for 24 h at 30 °C followed by two days at room temperature (RT), the cells were harvested by centrifugation. Each pellet was resuspended in 1 mL of lysis buffer [50 mM Tris-HCl, 300 mM NaCl, 0.1% v/v Triton X-100, 100 µM EDTA pH8.0, 0.5 mg/mL lysozime, 20 mM Imidazole, protease inhibitors (0.5 mM PMSF, 0.4 µg/mL leupeptin, 0.5 µg/mL aprotinin), and 5 µg/mL DNase and RNase]. The resuspended cells were lysed by sonication. The cell debris were removed by centrifugation at 4 °C for 20 min at 13,000 rpm. The protein concentration of the whole cell extracts was determined by the Bradford method [71], using the Bio-Rad protein assay kit (Bio-Rad Laboratories, Hercules, CA, USA). Equal amounts of protein (20 μg) were subjected to 13% SDS-PAGE and visualized by Western blotting using an anti-GFP antibody (# 600-101-215, Rockland, Limerik, PA, USA).

### 4.4. GFP-Fragment Reassembly Screening

*E. coli* BL21 (DE3) cells were co-transformed with the following compatible pairs of plasmids: pET11a-NfrGFP-Z and pMRBAD-Z-CfrGFP, pET11a-NfrGFP-BARD1, or pET11a-NfrGFP-UbcH5a and pMRBAD-BRCA1-CfrGFP (both as wild-type or mutant forms), pET11a-NfrGFP-BRCA2HD/OB1/OB2/OB3, or pMRBAD-BRCA2HD/OB1/OB2/OB3-CfrGFP (both as wild-type or mutant forms) and pMRBAD-DSS1-CfrGFP or pET11a-NfrGFP-DSS1, and screened for the occurrence of GFP-fragment reassembly, as previously described [14]. Fluorescence was observed after excitation with long-wave (365 nm) UV light combined with the short pass (SP) emission filter using a Syngene image capture system (SYNGENE, Cambridge, UK), as specified by the manufacturer.

### 4.5. Purification of the Reassembled GFP Complexes

The H_6_-NfrGFP-BARD1(or UbcH5a)/BRCA1-CfrGFP (both as wild-type or mutant forms) and H_6_-NfrGFP-BRCA2HD/OB1 (both as wild-type or mutant forms)/DSS1-CfrGFP complexes were purified from the soluble fraction of co-transformed *E. coli* strain BL21 (DE3) by IMAC using nickel nitrilotriacetic (Ni-NTA) agarose resin (QIAGEN, Hilden, Germany), according to the manufacturer’s instruction. The protein complexes were subjected to 13% SDS-PAGE and visualized by Western blotting using a polyclonal anti-GFP antibody (# 600-101-215, Rockland).

### 4.6. Protein Structures

The 3D crystallographic structures of the BRCA1-BARD1 heterodimeric complex and of BRCA2 in complex with the protein DSS1 were represented by the Swiss-PdbViewer tool (SPDBV, URL: http://www.expasy.org/spdbv/, accessed on 7 May 2015) [72] using the pdb as a template (protein data bank, URL: http://www.pdb.org, accessed on 7 May 2015) [73] files 1JM7 and 1MJE, respectively.

### 4.7. In Silico Analyses

The Alamut Visual version 2.10 (Interactive Biosoftware, Rouen, France) was used to evaluate the possible effect of examined VUS on mRNA transcript splicing.

## 5. Conclusions

The concordance between genetic data and the functional assays analyzing the BRCA1/UbcH5a and the BRCA2/DSS1 interactions, observed for the validation panels of previously classified variants, strongly suggests that the approaches described in the present study are able to differentiate between VUS that either inactivate or have no effect on *BRCA1* and *BRCA2* functions and support their utility in the assessment of the clinical relevance of VUS. Caution is warranted when the interpretation of VUS is based only on results from an assay focusing on a single specific biochemical activity. Therefore, these assays should be used in combination with other evidence when assessing variant pathogenicity, and the efficacy will be further verified using larger validation panels.

## Figures and Tables

**Table 1:** Variants of validation panels.

Gene	DNA Change	Protein Change	Domain	Motif	IARC Class ^a^	References/Criterion
**BRCA1**	c.53T>C	p.Met18Thr	RING finger	Helical bundle N	4	[6]
	c.65T>C	p.Leu22Ser	RING finger	Helical bundle N	5	[51]
	c.110C>A	p.Thr37Lys	RING finger	β-Sheet	5	[51]
	c.115T>C	p.Cys39Arg	RING finger	Zn^2+^/SiteII	5	[51]
	c.116G>A	p.Cys39Tyr	RING finger	Zn^2+^/SiteII	5	[52]
	c.122A>G	p.His41Arg	RING finger	Zn^2+^/SiteII	5	[53]
	c.130T>A	p.Cys44Ser	RING finger	Zn^2+^/SiteI	5	[51]
	c.131G>T	p.Cys44Phe	RING finger	Zn^2+^/SiteI	5	[52]
	c.131G>A	p.Cys44Tyr	RING finger	Zn^2+^/SiteI	5	[51]
	c.133A>C	p.Lys45Gln	RING finger		1	[51]
	c.181T>G	p.Cys61Gly	RING finger	Zn^2+^/SiteII	5	[54]
	c.191G>A	p.Cys64Tyr	RING finger	Zn^2+^/SiteII	5	[52]
	c.199G>T	p.Asp67Tyr	RING finger		1	[6]
**BRCA2**	c.7469T>C	p.Ile2490Thr	DBD	HD	1	Frequency >1% in outbred populations
	c.7544C>T	p.Thr2515Ile	DBD	HD	1	[55]
	c.7826G>A	p.Gly2609Asp	DBD	HD	4	[8,47]
	c.7878G>C	p.Trp2626Cys	DBD	HD	5	[6]
	c.7879A>T	p.Ile2627Phe	DBD	HD	5	[6]
	c.7940T>C	p.Leu2647Pro	DBD	HD	4	[46]
	c.7958T>C	p.Leu2653Pro	DBD	HD	5	[6,46]
	c.8063T>C	p.Leu2688Pro	DBD	OB1	4	[47]
	c.8149G>T	p.Ala2717Ser	DBD	OB1	1	[55]
	c.8165C>G	p.Thr2722Arg	DBD	OB1	5	[6,46]
	c.8167G>C	p.Asp2723His	DBD	OB1	5	[5,8]
	c.8168A>G	p.Asp2723Gly	DBD	OB1	5	[6,47,56]
	c.8187G>T	p.Lys2729Asn	DBD	OB1	1	[6,8]
	c.8243G>A	p.Gly2748Asp	DBD	OB1	5	[6,8]
	c.8324T>G	p.Met2775Arg	DBD	OB1	2	[52]

^a^ Class 1/2, non pathogenic/likely non pathogenic, respectively. Class 4/5, likely pathogenic/pathogenic, respectively. Abbreviations: DBD, DNA binding domain. HD, helical domain. OB1, oligonucleotide/oligosaccharide binding fold 1.

**Table 2:** List of selected *BRCA1* and *BRCA2* VUS.

Gene	DNA Change	Protein Change	Domain	Motif	Source	ClinVar
**BRCA1**	c.20_28del9	p.7-10delinsGln	RING finger		INT	VUS (1)
	c.43A>G	p.Ile15Val	RING finger	Helical bundle N	INT	NR
	c.44T>C	p.Ile15Thr	RING finger	Helical bundle N	INT	VUS (4)
	c.53T>A	p.Met18Lys	RING finger	Helical bundle N	ClinVar	VUS (2)
	c.70T>C	p.Cys24Arg	RING finger	Zn^2+^/SiteI	ClinVar	VUS (1); LP (1), P (1)
	c.79_80delTGinsGC	p.Cys27Ala	RING finger	Zn^2+^/SiteI	Synthetica	NR
	c.83T>C	p.Leu28Pro	RING finger		INT	VUS (4)
	c.93C>G	p.Ile31Met	RING finger		INT	LB (1), VUS (2)
	c.100C>T	p.Pro34Ser	RING finger		Rigshospitalet	VUS (2)
	c.107C>A	p.Ser36Tyr	RING finger	β-strand	ICO	VUS (5)
	c.110C>G	p.Thr37Arg	RING finger	β-strand	ClinVar	LP (1)
	c.115T>G	p.Cys39Gly	RING finger	Zn^2+^/SiteII	Rigshospitalet	VUS (1), LP (1), P (1)
	c.121_122delCAinsGC	p.His41Ala	RING finger	Zn^2+^/SiteII	Synthetic ^a^	NR
	c.124A>G	p.Ile42Val	RING finger		Clinvar	3 LB (1), VUS (3)
	c.139T>G	p.Cys47Gly	RING finger	Zn^2+^/SiteI	Clinvar	LP (1), P (6)
	c.143T>G	p.Met48Arg	RING finger	Central Helix	Rigshospitalet	NR
	c.143T>A	p.Met48Lys	RING finger	Central Helix	INT	NR
	c.190T>G	p.Cys64Gly	RING finger	Zn^2+^/SiteII	Clinvar	VUS (1), P (11)
	c.190T>C	p.Cys64Arg	RING finger	Zn^2+^/SiteII	INT	VUS (1), P (4)
	c.206_207delCCinsTG	p.Thr69Met	RING finger		INT	NR
	c.230C>T	p.Thr77Met	RING finger		INT	VUS (6)
	c.248T>G	p.Val83Gly	RING finger	Helical bundle C	ICO	NR
	c.293G>T	p.Gly98Val	RING finger		INT	NR
**BRCA2**	c.7505G>A	p.Arg2502His	DBD	HD	INT	B (1), LB (5), VUS (3)
	c.7628A>G	p.Tyr2543Cys	DBD	HD	INT	VUS (7)
	c.7636T>C	p.Ser2546Pro	DBD	HD	INT	VUS (1)
	c.7649T>C	p.Ile2550Thr	DBD	HD	Clinvar	B (1)
	c.7684T>C	p.Phe2562Leu	DBD	HD	INT	VUS (6)
	c.7698T>G	p.Asp2566Glu	DBD	HD	INT	NR
	c.7930A>G	p.Asn2644Asp	DBD	HD	INT	VUS (3)

^a^ [38]. Abbreviations: DBD, DNA binding domain. HD, helical domain, INT, Fondazione IRCCS Istituto Nazionale dei dei Tumori (Milan, Italy), ICO, Catalan Institute of Oncology (Hospitalet de Llobregat, Barcelona, Spain). Rigshospitalet (Copenhagen, Denmark). Clinvar, URL: https://www.ncbi.nlm.nih.gov/clinvar/. VUS, variants of uncertain significance. NR, not reported. LP, likely pathogenic. P, pathogenic. LB, likely benign. B, benign.

**Table 3:** *BRCA1* variants analyzed with different functional approaches.

Variants Group	DNA Change	Protein Change	Motif	Align-GVGD ^a^	IARC Class ^b^	BARD1 Binding ^d^	UbCH5a Binding ^d^	Ubiquitin Ligase Activity ^e,f^	Restoration of Radiation resistance ^f^	HDR ^g^	mESCs-Based Assays ^h^	Cisplatin Response ^h^	Small Colony Phenotype ^i^	Yeast Localization Phenotype i	Single strand Annealing ^l^	Centrosome Number ^m^	HDR Rescue ^n^		Functinal Effect ^o^
																	Pred	Func	
**Validation Panel**	c.53T>C	p.Met18Thr	Helical bundle N	C45	4	+ (also in c)	−	−	−	−	−	−	−	−	−	−	−	−	LOF
	c.65T>C	p.Leu22Ser	Helical bundle N	C65	5	−	−	ND	ND	ND	ND	ND	ND	ND	ND	ND	+	−	LOF
	c.110C>A	p.Thr37Lys		C65	5	+	−	ND	ND	ND	ND	ND	ND	ND	ND	ND	−	ND	LOF
	c.115T>C	p.Cys39Arg	Zn^2+^/SiteII	C65	5	−	−	−	ND	ND	ND	ND	ND	ND	ND	ND	−	ND	LOF
	c.116G>A	p.Cys39Tyr	Zn^2+^/SiteII	C65	5	−	−	−	−	−	ND	ND	−	ND	−	−	−	−	LOF
	c.122A>G	p.His41Arg	Zn^2+^/SiteII	C25	5	+	−	−	ND	−	ND	ND	ND	ND	−	−	+	−	LOF
	c.130T>A	p.Cys44Ser	Zn^2+^/SiteI	C65	5	−	−	ND	ND	ND	ND	ND	ND	ND	ND	ND	−	−	LOF
	c.131G>A	p.Cys44Tyr	Zn^2+^/SiteI	C65	5	−	−	ND	ND	ND	ND	ND	−	−	ND	ND	−	ND	LOF
	c.131G>T	p.Cys44Phe	Zn^2+^/SiteI	C65	5	−	−	−	ND	−	ND	ND	ND	ND	−	−	−	−	LOF
	c.133A>C	p.Lys45Gln		C0	1	+	+	ND	ND	ND	+	ND	ND	ND	ND	ND	+	+	FUNC
	c.181T>G	p.Cys61Gly	Zn^2+^/SiteII	C65	5	−	−	−	−	−	−	−	+	−	−	ND	−	−	LOF
	c.191G>A	p.Cys64Tyr	Zn^2+^/SiteII	C65	5	−	−	−	ND	ND	ND	ND	ND	ND	ND	ND	−	ND	LOF
	c.199G>T	p.Asp67Tyr		C0	1	+	+	+	ND	+	+	+	ND	ND	+	ND	+	+	FUNC
**VUS**	c.20_28del9	p.7-10delinsGln		NA	NA	−	−	ND	ND	ND	ND	ND	ND	ND	ND	ND	ND	ND	ND
	c.43A>G	p.Ile15Val	Helical bundle N	C15	NA	−	+	ND	ND	ND	ND	ND	ND	ND	ND	ND	+	ND	FUNC
	c.44T>C	p.Ile15Thr	Helical bundle N	C65	NA	+ (also in ^a^)	−	−	ND	ND	ND	ND	ND	ND	ND	ND	−	−	LOF
	c.53T>A	p.Met18Lys	Helical bundle N	C55	NA	− (also in ^a^)	−	−	ND	ND	ND	ND	ND	ND	ND	ND	−	ND	ND
	c.70T>C	p.Cys24Arg	Zn^2+^/SiteI	C65	NA	−	−	−	−	−	ND	ND	ND	ND	−	−	−	−	LOF
	c.79_80delTGinsGC	p.Cys27Ala	Zn^2+^/SiteI	C65	NA	−	−	ND	ND	−	ND	ND	ND	ND	ND	−	−	−	ND
	c.83T>C	p.Leu28Pro		C65	NA	+	−	+/−	ND	ND	ND	ND	ND	ND	ND	ND	+	−	LOF
	c.93C>G	p.Ile31Met		C0	NA	+	+	+	+	+	ND	ND	ND	ND	+	ND	+	+	FUNC
	c.100C>T	p.Pro34Ser		C65	NA	−	−	ND	ND	ND	ND	ND	ND	ND	ND	ND	+	ND	LOF
	c.107C>A	p.Ser36Tyr	β-strand	C15	NA	+	−	ND	ND	ND	ND	ND	ND	ND	ND	ND	+	ND	ND
	c.110C>G	p.Thr37Arg		C65	NA	−	−	−	−	−	ND	ND	ND	ND	−	+	−	−	LOF
	p c.115T>G	p.Cys39Gly	Zn^2+^/SiteII	C65	NA	−	−	ND	ND	ND	ND	ND	ND	ND	ND	ND	−	−	LOF
	c.121_122delCAinsGC	p.His41Ala	Zn^2+^/SiteII	C65	NA	−	−	ND	ND	ND	ND	ND	ND	ND	ND	ND	+	ND	ND
	c.124A>G	p.Ile42Val		C0	NA	+	+	+	+	+	ND	ND	ND	ND	+	−	+	+	FUNC
	c.139T>G	p.Cys47Gly	Zn^2+^/SiteI	C65	NA	−	−	−	ND	ND	ND	ND	ND	ND	ND	ND	−	−	LOF
	c.143T>G	p.Met48Arg	Central Helix	C35	NA	−	−	ND	ND	ND	ND	ND	ND	ND	ND	ND	−	−	LOF
	c.143T>A	p.Met48Lys	Central Helix	C45	NA	+	−	ND	ND	ND	ND	ND	ND	ND	ND	ND	+	ND	LOF
	q c.190T>G	p.Cys64Gly	Zn^2+^/SiteII	C65	NA	−	−	−	ND	−	−	ND	ND	ND	−	ND	−	ND	LOF
	r c.190T>C	p.Cys64Arg	Zn^2+^/SiteII	C65	NA	−	−	ND	ND	ND	ND	ND	ND	ND	ND	ND	−	ND	LOF
	c.206_207delinsTG	p.Thr69Met		C65	NA	−	−	ND	ND	ND	ND	ND	ND	ND	ND	ND	+	ND	ND
	c.230C>T	p.Thr77Met		C0	NA	+	+	−	ND	ND	ND	ND	ND	ND	ND	ND	+	ND	FUNC
	c.248T>G	p.Val83Gly	Helical bundle C	C0	NA	−	+	ND	ND	ND	ND	ND	ND	ND	ND	ND	+	ND	FUNC
	c.293G>T	p.Gly98Val		C65	NA	+	−	ND	ND	ND	ND	ND	ND	ND	ND	ND	+	ND	LOF

^a^ Prior probability of pathogenicity based on Align GVGD (URL: http://agvgd.iarc.fr/agvgd_input.php): C0 (1%), C15, C25 (29%), C3, C4, C55 (66%), C65 (81%); ^b^ Class 1/2, non-pathogenic/likely non-pathogenic, respectively. Class 4/5, likely pathogenic/ pathogenic, respectively. ^c^ [14], ^d^ This study, ^e^ [37], ^f^ [30], ^g^ [38], ^h^ [39], ^i^ [40], ^l^ [41], ^m^ [42]: +, the missense variant has no effect on BRCA1 function. −, the missense variant disrupts BRCA1 function. ^n^ [43]: −, HDR rescue prediction and/or functional score <0.53, +, HDR rescue prediction and/or functional score >0.53, assuming 0.53 as the threshold for classifying a variant as functional (HDR prediction score is calculated by combining the Ubiquitin ligase activity and BARD1 binding functional scores). ^o^ [44]: functional classification based on the saturation genome editing (SGE) score. ^p^ Predicted splicing affecting the variant. ^q^ splicing affecting variant. ^r^ [15]. Abbreviations: NA, not applicable. ND, not done. HDR, homology directed recombination. mESCs, mouse embryonic stem cells. Pred, predicted HDR rescues score. Func, functional: LOF, loss of function.

**Table 4:** *BRCA2* variants analyzed with different functional approaches.

Variants Group	DNA Change	Protein Change	Domain	Motif	Align-GVGD ^a^	IARC Class ^b^	BRCA2/DSS1 Binding ^c^	HDR ^d^	Centrosome Amplification ^e^	mESCs-Based Cell-Viability ^f,g^	mESCs-Based HDR ^g^
**Validation panel**	c.7469T>C	p.Ile2490Thr	DBD	HD	C0	1	+	ND	ND	+	ND
	c.7544C>T	p.Thr2515Ile	DBD	HD	C0	1	+	+/−	+/−	+	+
	c.7826G>A	p.Gly2609Asp	DBD	HD	C65	4	−	−	ND	+	−
	c.7878G>C	p.Trp2626Cys	DBD	HD	C65	5	−	−	ND	−	ND
	c.7879A>T	p.Ile2627Phe	DBD	HD	C15	5	−	−	−	−	ND
	c.7940T>C	p.Leu2647Pro	DBD	HD	C65	4	−	−	−	ND	ND
	c.7958T>C	p.Leu2653Pro	DBD	HD	C65	5	−	−	−	ND	ND
	c.8063T>C	p.Leu2688Pro	DBD	OB1	C65	4	−	−	ND	−	ND
	c.8149G>T	p.Ala2717Ser	DBD	OB1	C0	1	+	ND	ND	+	+
	c.8165C>G	p.Thr2722Arg	DBD	OB1	C65	5	−	−	−	−	ND
	c.8167G>C	p.Asp2723His	DBD	OB1	C65	5	−	−	−	−	ND
	c.8168A>G	p.Asp2723Gly	DBD	OB1	C65	5	−	−	−	−	ND
	c.8187G>T	p.Lys2729Asn	DBD	OB1	C0	1	+	+	+	+	+
	c.8243G>A	p.Gly2748Asp	DBD	OB1	C65	5	−	−	−	−	ND
	c.8324T>G	p.Met2775Arg	DBD	OB1	C0	2	+	ND	ND	ND	ND
**VUS**	c.7505G>A	p.Arg2502His	DBD	HD	C0	NA	+	ND	ND	ND	ND
	c.7628A>G	p.Tyr2543Cys	DBD	HD	C15	NA	+	ND	ND	ND	ND
	c.7636T>C	p.Ser2546Pro	DBD	HD	C0	NA	+	ND	ND	ND	ND
	c.7649T>C	p.Ile2550Thr	DBD	HD	C25	NA	+	ND	ND	ND	ND
	c.7684T>C	p.Phe2562Leu	DBD	HD	C15	NA	−	−	ND	ND	ND
	c.7698T>G	p.Asp2566Glu	DBD	HD	C0	NA	+	ND	ND	ND	ND
	c.7930A>G	p.Asn2644Asp	DBD	HD	C0	NA	+	ND	ND	ND	ND

^a^ Prior probability of pathogenicity based on Align GVGD (URL: http://agvgd.iarc.fr/agvgd_input.php): C0 (1%), C15, C25 (29%), C3, C4, C55 (66%), C65 (81%). ^b^ Class 1/2: non-pathogenic/likely non-pathogenic and class 4/5: pathogenic/likely pathogenic. ^c^ This study ^d^ [45,46,47,48], ^e^ [45,46], ^f^ [49], ^g^ [50]: “+”: the missense variant has no effect on BRCA2 function. “−“: the missense variant disrupts BRCA2 function. Abbreviations: DBD, DNA binding domain. HD, helical domain. OB1, oligonucleotide/oligosaccharide binding fold 1. NA: not applicable. ND: not done. HDR: homology directed repair. mESCs: mouse embryonic stem cell.

## Supplementary Materials

The following are available online at http://www.mdpi.com/2072-6694/11/2/151/s1, Figure S1: Fluorescence complementation through GFP-reassembly assay depends on fusion orientation of DSS1 and BRCA2 fragments, Figure S2: Purification of the BARD1/BRCA1 reassembled complexes by the IMAC Method, Figure S3: Purification of the UbCH5a/BRCA1 reassembled complexes by the IMAC Method, Figure S4: Purification of the DSS1/BRCA2 reassembled complexes by the IMAC method.
