# Supplementary material for: GFP-Fragment Reassembly Screens for the Functional Characterization of Variants of Uncertain Significance in Protein Interaction Domains of the BRCA1 and BRCA2 Genes
Source: Cancers (Basel). 2019 Jan 28;11(2):151. doi: 10.3390/cancers11020151 (PMC6406614; doi:10.3390/cancers11020151)
Supplement: Supplementary file 1 [file cancers-11-00151-s001.pdf]

Supplementary Materials: GFP-Fragment Reassembly Screens for the Functional Characterization of Variants of Uncertain Significance in Protein Interaction Domains of the *BRCA1* and *BRCA2* Genes

Laura Caleca, Mara Colombo, Thomas van Overeem Hansen, Conxi Lázaro, Siranoush Manoukian, Michael T. Parsons, Amanda B. Spurdle and Paolo Radice

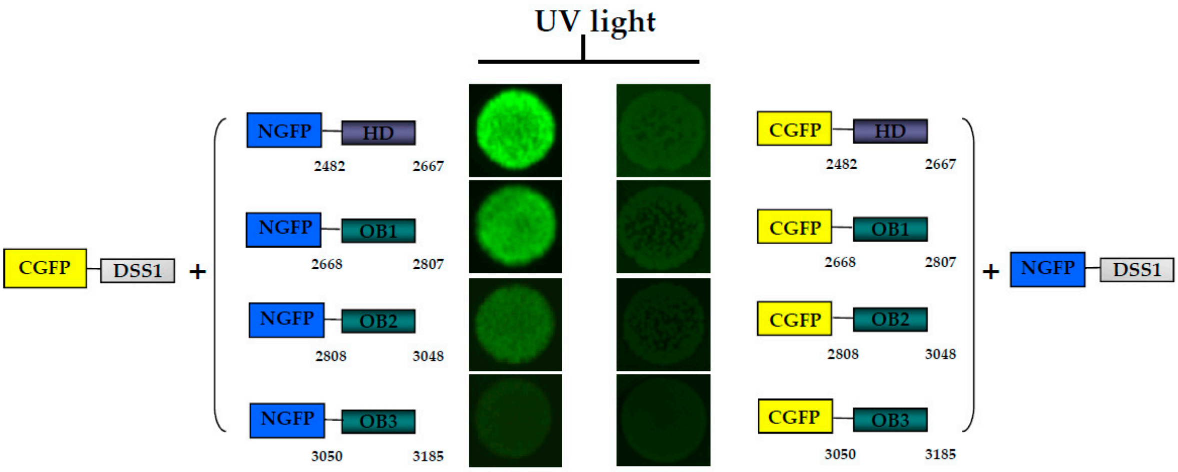

Figure S1. Fluorescence complementation through GFP-reassembly assay depends on fusion orientation of DSS1 and BRCA2 fragments. Fluorescence was recovered after 24 h of growth at 30 °C followed by 2 days of incubation at RT. All pictures were taken with the same digital camera (long-wave UV light, 365 nm).

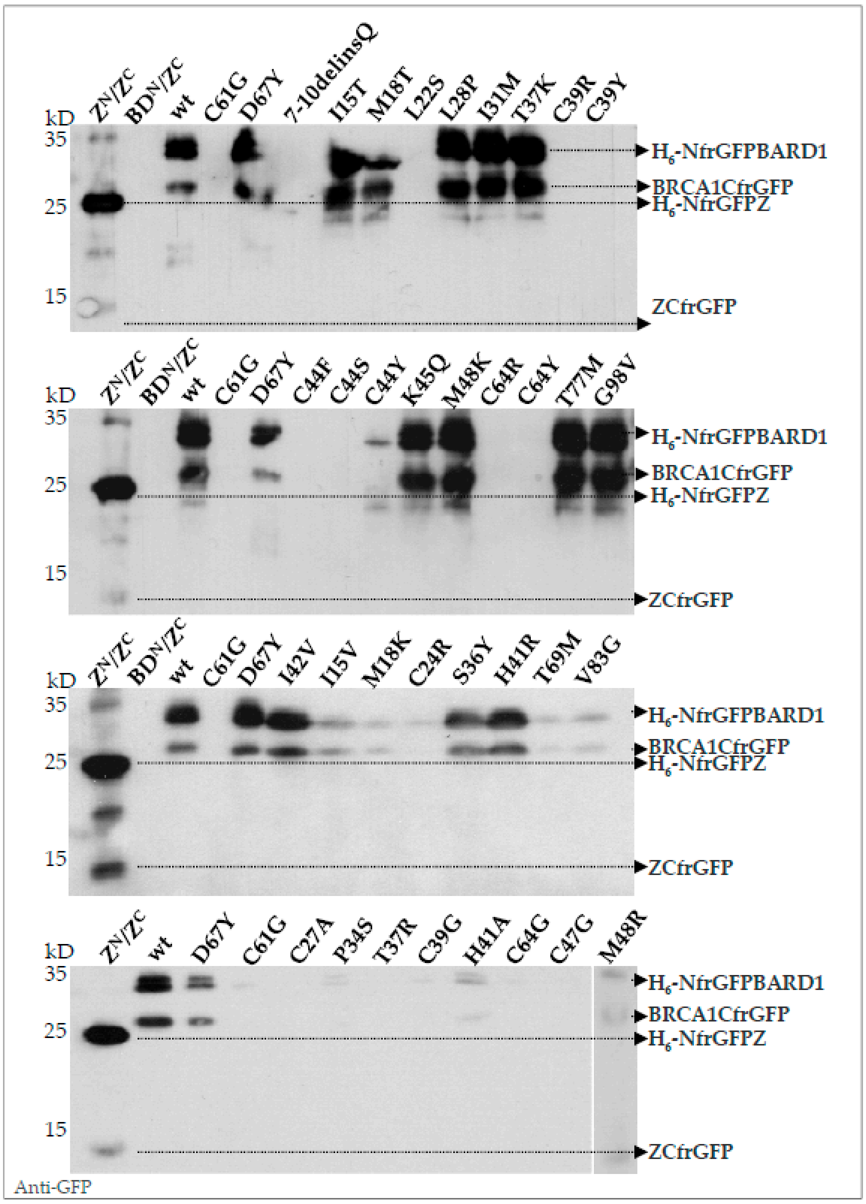

Figure S2. Purification of the BARD1/BRCA1 reassembled complexes by IMAC method. ZN/ZC, BDN/BR1C (wt) and D67Y were included as positive controls. BDN/ZC and C61G were included as negative control. The molecular masses are indicated on the left. [ZN, H_6-NfrGFPZ; ZC, ZCfrGFP; BDN, H_6-NfrGFPBARD1].

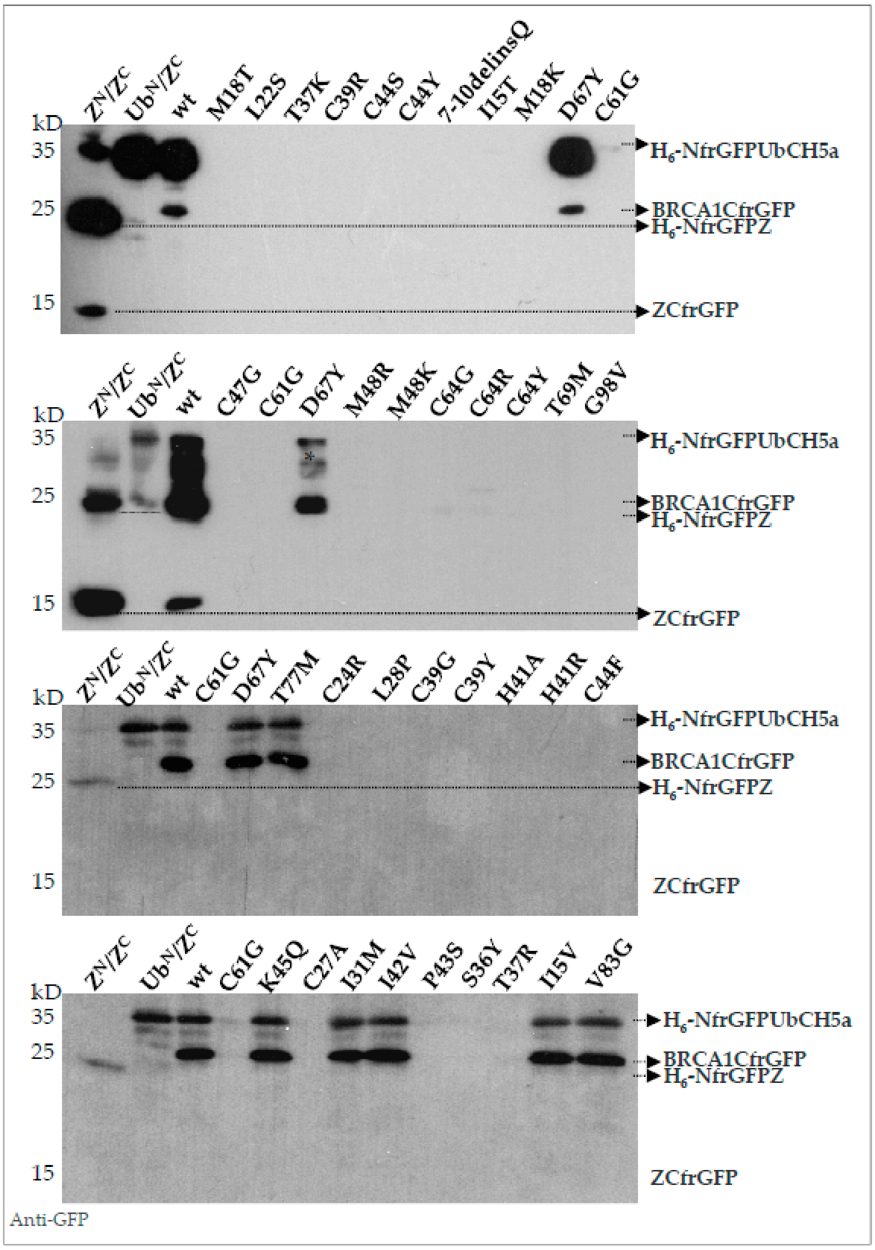

Figure S3. Purification of the UbCH5a/BRCA1 reassembled complexes by IMAC method. ZN/ZC, UbN/BR1C (wt) and D67Y or K45Q were included as positive controls. UbN/ZC and C61G were included as negative control. The molecular masses are indicated on the left. [ZN, H6-NfrGFPZ; ZC, ZCfrGFP; UbN, H6-NfrGFPUbCH5a].

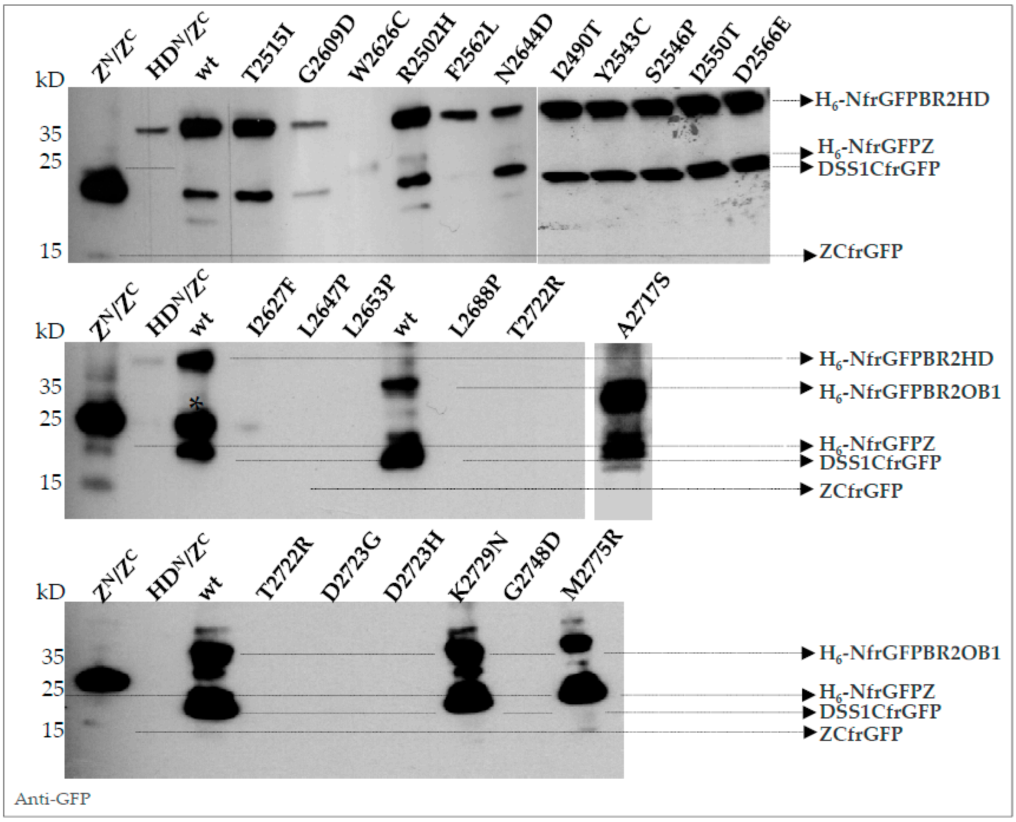

Figure S4. Purification of the DSS1/BRCA2 reassembled complexes by IMAC method. ZN/ZC and DSS1C with the wild type form of each of the BRCA2 motif (wt) were included as positive controls. HDN/ZC was included as negative control. The molecular. masses are indicated on the left. [ZN, H6-NfrGFPZ; ZC, ZCfrGFP; BR2HDN, H6-NfrGFPBRCA2HD; * non-specific band].
